# Scrotal migration of a ventriculoperitoneal shunt in an adult. A case report and literature review

**DOI:** 10.1016/j.bas.2022.100898

**Published:** 2022-06-06

**Authors:** Mohamed Khoudir, Lauren Harris, Sebastian M. Toescu, Babar Vaqas

**Affiliations:** Department of Neurosurgery, Queen's Hospital, Rom Valley Way, Romford, Essex, RM7 0AG, UK

**Keywords:** Ventriculoperitoneal shunt, Surgical complication, Scrotum, Revision, Neurosurgery

## Abstract

**Introduction:**

Scrotal migration of a ventriculoperitoneal shunt (VPS) catheter is a rare complication of VPS. Scrotal migrations usually manifest in the first year post-operatively, usually in the pediatric population, due to processus vaginalis patency and increased abdominal pressure.

**Research question:**

To review cases of scrotal migration of a VPS catheter that occur in the adult population, and its recommended management.

**Material and methods:**

A case report and review of the literature.

**Results:**

A 75-year-old male with a ventriculoperitoneal shunt for normal pressure hydrocephalus, presented with testicular swelling. Imaging revealed that the distal shunt catheter had migrated into his scrotum. He required an emergency shunt revision, with a truncation of the catheter, and involvement of the general surgical team for hernia management. He remained well at one year follow-up.

**Discussion and conclusion:**

To the best of our knowledge, this is the fifth case in an adult. This case serves as a reminder to take a thorough clinical history, imaging of the entire VPS pathway, and to consider unusual reasons for VPS failures. Emergency intervention for distal shunt revision is required to prevent further neurological or urological morbidity. Treatment includes not only catheter revision and reinsertion, but the catheter should be truncated, to avoid testicular migration recurrence. Hernia repair can be done either as an emergency or elective case, depending on the patient's clinical status and presentation.

## Highlights

• Ventriculoperitoneal catheter migration to the scrotum can occur in adults, as well as more commonly in paediatrics.
• This is the fifth adult case of scrotal distal cather shunt migration.
• This can cause a hydrocele and result in shunt failure.
• This can necessitate shunt revision and involvement of the general surgical team.
• In shunt failure, or scrotal swelling, thorough work-up must be done to diagnose rare, potentially fatal, conditions.

## 1 Introduction

Ventriculoperitoneal shunt (VPS) surgery is a common neurosurgical procedure whose complications are well documented. These include infection, obstruction, over or under-drainage and proximal or distal catheter migration (to the abdominal wall, diaphragm, heart, lungs, and rarely the testis). Although distal catheter migration usually has minor and benign consequences, as the tubing often remains within the peritoneal cavity, it can cause a symptomatic shunt malfunction, testicular torsion, or perforations, all of which are debilitating and potentially fatal disorders (Paff et al., 2018). 50 cases of distal catheter migration to the scrotum have been reported so far in the literature (all languages, no date limitation). Most of the cases presented in the pediatric age group due to a patent processus vaginalis, with only 4 cases reported in adults. Our case is the fifth known case documented in an adult worldwide (Perret et al., 2021).

## 2 Case presentation

**Fig. 1:**
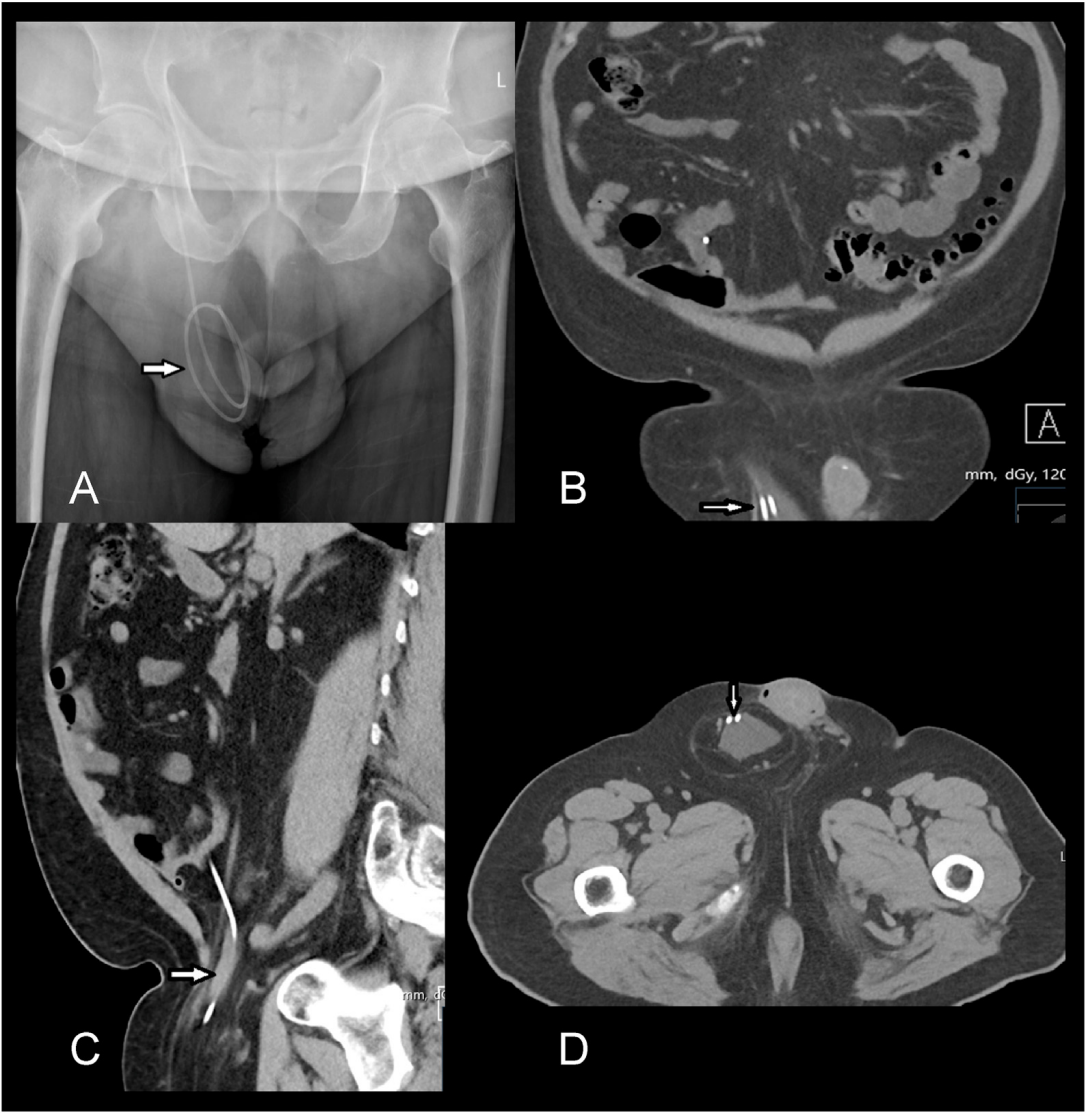
**A**, Preoperative AP abdominal radiograph showing the coiled distal shunt catheter inside the right scrotum (white arrow). **B**, coronal, **C**, sagittal and **D**, axial slices of abdominal CT scan showing distal shunt catheter in the hernia sac entering the scrotum.

**Fig. 2:**
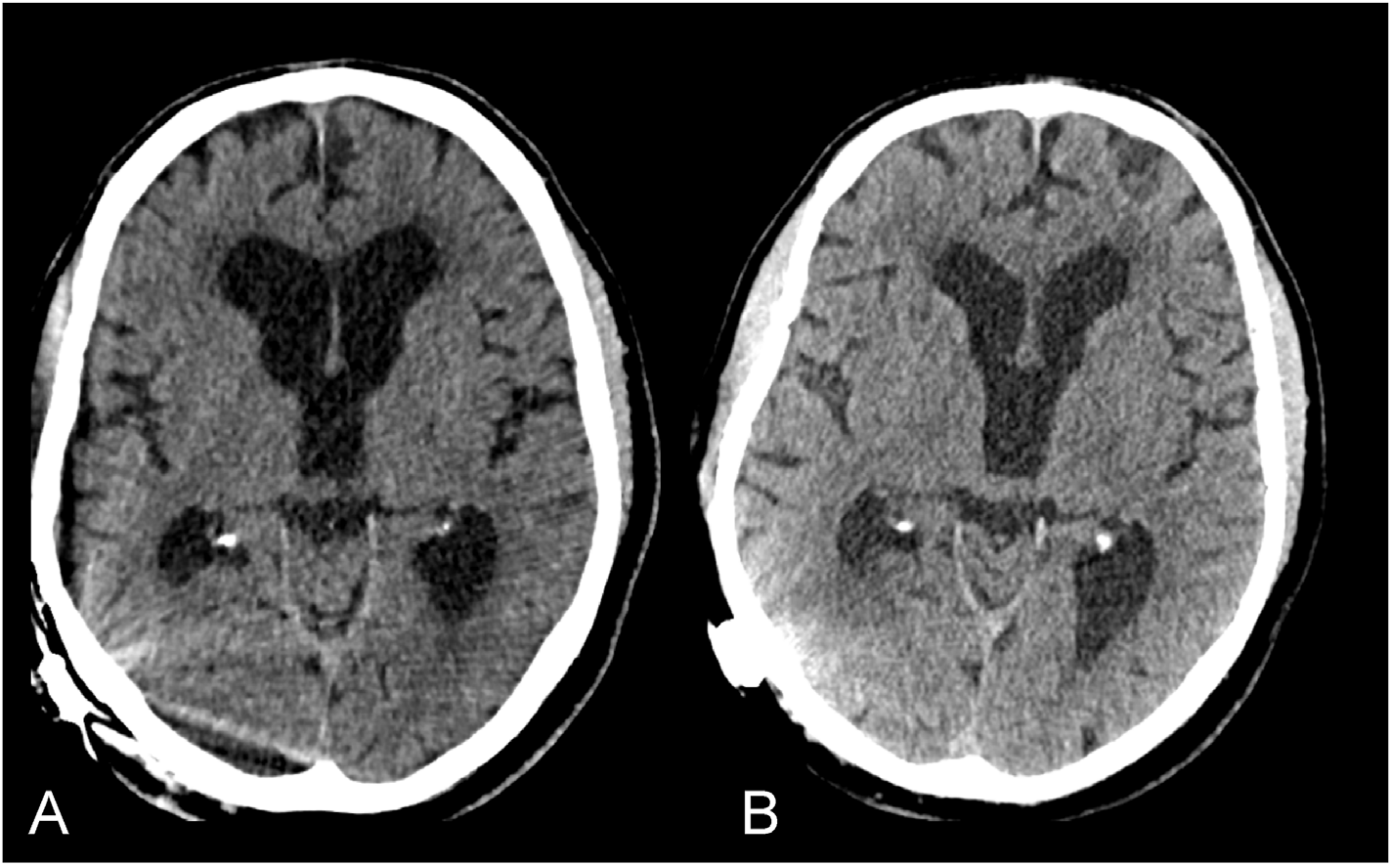
**A**, CT head immediately after insertion of the VPS. **B**, CT head upon presentation with hydrocoele, 4 months after VPS insertion, denoting unchanged ventricular calibre.

A 75 years old right-handed man underwent a programmable ventriculoperitoneal shunt (VPS) insertion for a confirmed diagnosis of normal pressure hydrocephalus (NPH) after serial lumbar punctures and neurological assessment. There were no immediate complications and post-operative shunt series confirmed correct placement of the distal catheter. He had an improvement in his gait and cognition. Four months postoperatively, the patient developed right sided testicular swelling which progressively increased and became painful over a week. The patient was systemically well with no change neurologically. He did not have any signs or symptoms of headache, vomiting, visual problems, gait disturbance, memory, or incontinence. Plain radiographs of the abdomen (Fig. 1A) showed the presence of the distal VPS catheter in his right hemiscrotum. Computerized tomography (CT) scan of the abdomen (Fig. 1B–D) confirmed a coiled ventriculoperitoneal catheter which had migrated inside an inguinal hernia. A hypodense fluid collection in the right scrotal area was discovered, along with coiled hyperdensity inside the mass, indicating catheter insertion into the scrotum. CT scan imaging of the head showed similar ventricular caliber to that on VPS insertion (Fig. 2).

**Fig. 3:**
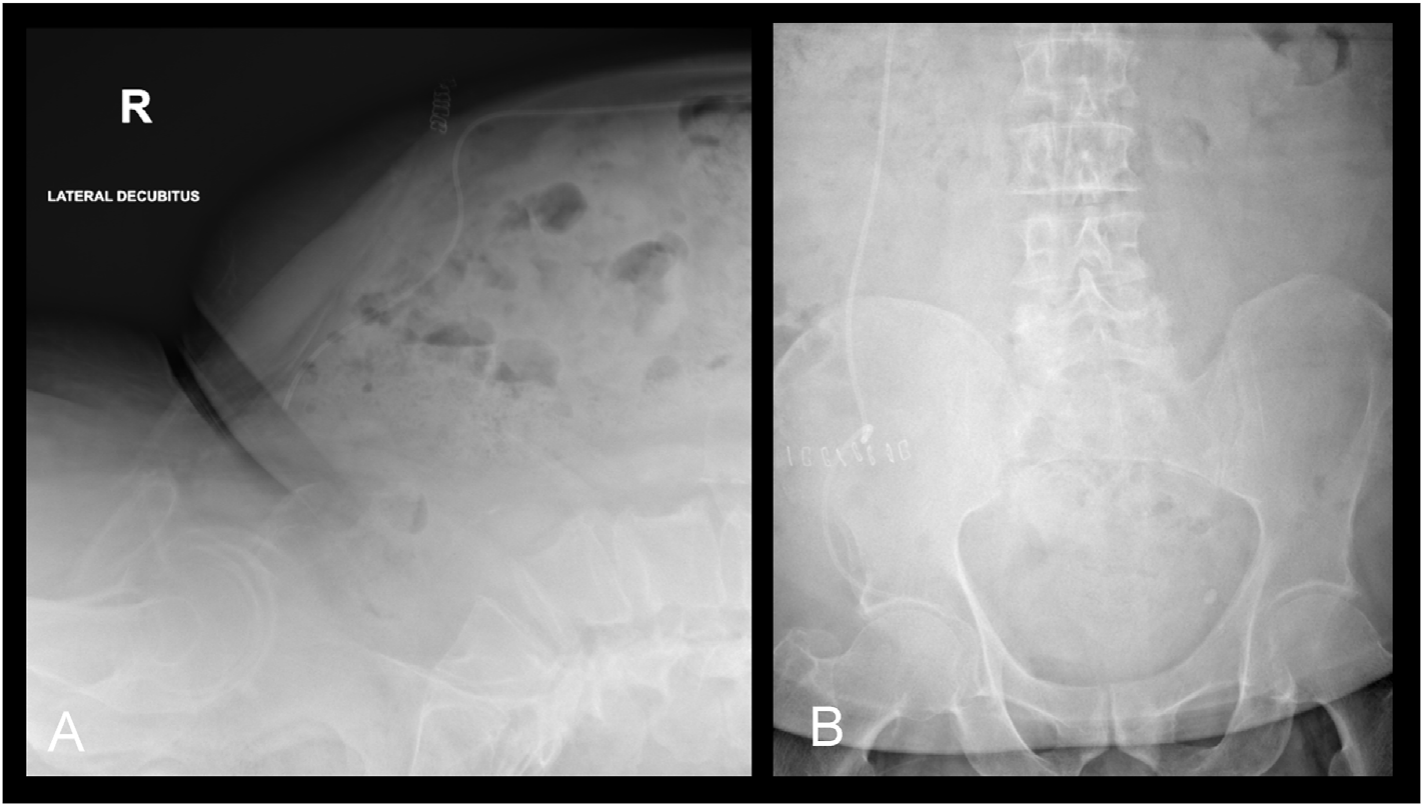
Postoperative radiographs in lateral (**A**) and anteroposterior (**B**) projections showing truncation and adequate placement of the distal shunt catheter.

The patient was admitted under neurosurgery and underwent an emergency VPS distal catheter revision under general anaesthetic, via the previous upper quadrant abdominal incision. The distal catheter was located and truncated to 20 cm length and reinserted into peritoneal cavity. Post-operative abdominal x-rays showed satisfactory location of the distal catheter in the peritoneal cavity (Fig. 3). The patient was discharged on day one post-operatively with no further complications.

He was seen by the general surgery team. His hernia was completely reducible with no evidence of obstruction, with a positive cough impulse. On outpatient review, he had no evidence of scrotal oedema or epididymo-orchitis, and he was listed for an elective hernia repair for hernia related pain. He remains well at one-year follow-up.

## 3Literature review and discussion

Peritoneal shunt catheter migration into the scrotum remains a rare complication of VPS insertion. This is the fifth reported case of scrotal migration of a VPS in an adult. Its incidence has been estimated at less than 1% (Bawa et al., 2017). During embryologic development, the processus vaginalis arises in both sexes as the peritoneum evaginates at the inguinal canal. The testes move from the posterior abdomen to the internal inguinal ring in males by 28 weeks of pregnancy, and by 32 weeks, the testes have entered the scrotum. The uterus' round ligament travels through the inguinal canal and terminates in the labia majora in females (Moore et al., 2018). The processus vaginalis becomes obliterated in the majority of the population over time. It is present in 90% at birth, 50–60% at the age of one, 40% between the ages of two and sixteen, and 15–30% in older persons at autopsies. The presence of the patent processus vaginalis together with small peritoneal cavity in pediatrics creates a pathway for distal catheter migration into the scrotum (Grosfeld and Cooney, 1974). In most instances, testicular migration of distal shunt catheter occurred in pediatric patients, mostly during infancy and the first six months following VPS shunt implantation (Mohammadi et al., 2012).

A total of 48 cases of this complication have been reported in the literature according to a recent systematic review (Hauser et al., 2021). The majority of the cases (93.75%) occurred in pediatric patients and only 3 cases occurred in adults. The review recommended management with a manual reposition maneuver in cases of short-lasting intact shunt tubing in the scrotum. The shunt tubing can often be manipulated into the peritoneal cavity and the hydroceles decreased by mild pull on the scrotal skin and push on the hernia, as with other hernias. This always failed in circumstances of long-standing or disconnected coiled tubing (Ricci et al., 2016).

A literature review was performed on December 11, 2021 on PubMed, Google Scholar and Medline using the search terms ‘ventriculoperitoneal’, ‘shunt’ and ‘scrotum’ which yielded related, cited, and citing publications. No limitations were placed on publication date or language of publication. VPS migration to the scrotum has been documented in 50 cases in the literature.

A summary of all adult cases of scrotal migration of a VPS is shown in Table 1, our case is the fifth worldwide. The first adult distal VPS scrotal migration was reported by Rehm et al. in 1997, in a 46 years old male four years post VPS insertion (Rehm et al., 1997). Whilst still draining, the catheter had migrated through the right inguinal canal and pierced the scrotal skin resulting in shunt infection. The distal end of the peritoneal catheter was drawn out via the scrotum through an incision made at the site of the earlier mini-laparotomy.

**Table 1:** Summary of adult cases with distal shunt catheter scrotal migration.

Cases	Author, year	Age	Site	Indication for VPS	Clinical picture	CT image	Time to presentation after shunting	Surgical intervention	Hernia repair
1	Rehm et al., 1997 (Rehm et al., 1997)	46	Right	Intraventricular tumour causing hydrocephalus	Testicular perforation and infection ​+ ​disturbed conscious level	Brain CT did not show any changes in the ventricular system	4 years	Mini-laparotomy ​+ ​peritoneal catheter divided and the distal end pulled out through the testis, infection treated and then new VP shunt	No hernia
2	Lee et al., 2015 (Lee et al., 2015)	65	Right	Normal pressure hydrocephalus	Only Scrotal swelling (hydrocele)	Not mentioned	7 days	Distal catheter trimming via laparoscopy done by general surgery staff	Not mentioned
3	Foster et al., 2017 (Foster et al., 2019)	27	Right	Neonatal post-haemorrhagic hydrocephalus	Scrotal swelling (hydrocele) ​+ ​papilledema ​+ ​proximal shunt catheter disconnected	A right parietal ventricular catheter was found disconnected from the shunt valve	27 years	Proximal catheter revision ​+ ​elective Hernia repair (at time of Surgery, distal catheter wasn't in anymore)	Elective
4	Perret et al., 2021 (Perret et al., 2021)	22	Right	Not mentioned	Scrotal swelling (hydrocele) ​+ ​disturbed conscious level	Acute hydrocephalus	6 years	VP shunt revision surgery	Emergency open inguinal hernia surgical repair.
5	Khoudir et al., 2022 (our case)	75	Right	Normal pressure hydrocephalus	Only Testicular swelling	No hydrocephalus	4 months	Distal catheter shortening	Elective

The second case was reported by Lee et al., 2015 in a 65-year-old man who had a VPS for NPH (Lee et al., 2015). He developed right testicular swelling on post-operative day seven, for which an ultrasound revealed a hydrocele and the presence of a distal catheter in the scrotum. On the ninth post-operative day, distal catheter trimming via laparoscopy was successfully performed by general surgery, with post-operative imaging revealing a successful placement of the distal catheter in the peritoneal cavity.

The third case by Foster et al. 2017 was in a 26-year-old whose scrotal migration presented with progressive positional headaches, vomiting, and neck stiffness for three weeks (Foster et al., 2019). Eye examination revealed bilateral papilledema. He was treated with a *de-novo* right parietal ventricular catheter, and an elective hernia surgery, during which the distal catheter was not used. The fourth case by Perret et al., 2021 was in a 22-year-old man who presented with acute hydrocephalus six years post VPS insertion. He underwent VP shunt revision surgery and open inguinal hernia repair surgery (Perret et al., 2021). The most recent case by Javed et al., 2021 was in a 5 month-old who had a VPS for a communicating hydrocephalus, shunt was revised on the 4th day due to testicular swelling and distal shunt migration (Javed et al., 2021). We believe that our case is the fifth adult recognized case of this shunt complication entity all over the word.

In all cases, the right-sided scrotum was involved, which can be explained by the fact that the right testicle descends later than the left. Repositioning of the distal catheter and processus vaginalis closure are two of the most typical treatments. The CSF flow into the patent processus vaginalis can provide a trough effect, pulling the shunt tip into the trough center (Kwok et al., 1989). There do not appear to be any predisposing factors to the development of this rare complication, except of course, the presence of herniae. This case, and other related reports, serve as a reminder of the importance of a thorough patient assessment prior to VPS insertion. In particular, examination of the abdomen to ascertain previous surgical intervention, or the presence of anterior abdominal wall deficits, is central in the pre-operative workup. The authors do not propose routine pre-operative imaging of the abdomen, except in cases where herniae have been elicited.

## 4 Conclusion

Scrotal migration of peritoneal catheters is a rare complication of VPS. It is more likely to occur in children, due to the patent processus vaginalis, than in adults. This is the fifth reported adult case, most of which have occurred in the presence of a coexistent inguinal hernia. This case serves as a reminder of the importance of to undertake a thorough patient assessment, post-operative imaging of the entire VPS pathway and consideration of unusual reasons for VPS failures. Emergency intervention for distal shunt revision is required to prevent further neurological or urological morbidity. Treatment includes not only catheter revision and reinsertion, but the catheter should be truncated, to avoid testicular migration recurrence. Hernia repair can be done either as an emergency or elective case depending patient's clinical status and presentation. Close consultation with general surgery teams for the consideration of a hernia repair should be the standard of care.

## Informed consent

Written informed consent for submission of the paper was obtained from the patient.

## Disclosure statement

The author declares no conflict of interest.

This research received no external financial or non-financial support.

There are no additional relationships to disclose.

There are no additional activities to disclose.

## Ethical approval

Patient was consented for approval for writing the case and publication.

## Contributors

All authors have contributed to the article and have approved the final article.

## Declaration of competing interest

The authors declare that they have no known competing financial interests or personal relationships that could have appeared to influence the work reported in this paper.
